# Chitosan microparticles loaded with yeast-derived PCV2 virus-like particles elicit antigen-specific cellular immune response in mice after oral administration

**DOI:** 10.1186/1743-422X-11-149

**Published:** 2014-08-20

**Authors:** Sergio A Bucarey, Myriam Pujol, Joaquín Poblete, Ignacio Nuñez, Cecilia V Tapia, Andrónico Neira-Carrillo, Jonatán Martinez, Oliver Bassa

**Affiliations:** Centro Biotecnológico Veterinario, Biovetec. Departamento de Ciencias Biológicas Animales, Facultad de Ciencias Veterinarias y Pecuarias, Universidad de Chile, La Pintana, Santiago, Chile; Center for Advanced Interdisciplinary Research in Materials (CIMAT) and Laboratory of Functionalized Polymers and Biomolecules (Polyforms), Departamento de Ciencias Biológicas Animales, Facultad de Ciencias Veterinarias y Pecuarias, Universidad de Chile, La Pintana, Santiago, Chile; Programa de Microbiología y Micología. Instituto de Ciencias Biomédicas, ICBM, Facultad de Medicina, Universidad de Chile, Independencia, Santiago, Chile; Biotechnological Veterinary Center, Biovetec. Departamento de Ciencias Biológicas Animales, Facultad de Ciencias Veterinarias y Pecuarias, Universidad de Chile, Santa Rosa 11735, La Pintana, Santiago, Chile

**Keywords:** PCV2, Chitosan microparticles, Yeast-derived VLPs, Oral delivery, Lymphocyte proliferation

## Abstract

**Background:**

Porcine circovirus type 2 (PCV2)-associated diseases are a major problem for the swine industry worldwide. In addition to improved management and husbandry practices, the availability of several anti-PCV2 vaccines provides an efficient immunological option for reducing the impact of these diseases. Most anti-PCV2 vaccines are marketed as injectable formulations. Although these are effective, there are problems associated with the use of injectable products, including laborious and time-consuming procedures, the induction of inflammatory responses at the injection site, and treatment-associated stress to the animals. Oral vaccines represent an improvement in antigen delivery technology; they overcome the problems associated with injection management and facilitate antigen boosting when an animals’ immunity falls outside the protective window.

**Methods:**

Chitosan microparticles were used as both a vehicle and mucosal adjuvant to deliver yeast-derived PCV2 virus-like particles (VLPs) in an attempt to develop an oral vaccine. The physical characteristics of the microparticles, including size, Zeta potential, and polydispersity, were examined along with the potential to induce PCV2-specific cellular immune responses in mice after oral delivery.

**Results:**

Feeding mice with PCV2 VLP-loaded, positively-charged chitosan microparticles with an average size of 2.5 μm induced the proliferation of PCV2-specific splenic CD4^+^/CD8^+^ lymphocytes and the subsequent production of IFN-γ to levels comparable with those induced by an injectable commercial formulation.

**Conclusion:**

Chitosan microparticles appear to be a safe, simple system on which to base PCV2 oral vaccines. Oral chitosan-mediated antigen delivery is a novel strategy that efficiently induces anti-PCV2 cellular responses in a mouse model. Further studies in swine are warranted.

## Background

Porcine circovirus-associated diseases (PCVAD) are a major problem affecting the productivity of the swine industry, resulting in considerable losses worldwide [1].

Porcine circovirus type 2 (PCV2) is thought to be the major causative agent of post-weaning multisystemic wasting syndrome (PMWS), a disease characterized by severe immunosuppression in the porcine host. The latest evidence suggests that PCV2-induced immune disorders are caused by silencing plasmacytoid dendritic cell responsiveness to pathogen-associated danger signals [2].

Consequently, PCV2 is also associated with many other conditions, including respiratory disease complex, reproductive failure, porcine dermatopathy and nephropathy syndrome (PDNS), congenital tremor, necrotizing tracheitis, and exudative epidermitis [3]. These diseases are known as PCVAD or PCV2-associated diseases, a name that the American Association of Swine Veterinarians (AASV) uses to group together all diseases attributed to PCV2, including PMWS [4].

In addition to improved management and husbandry (e.g., better hygiene, less overcrowding, and better ventilation), anti-PCV2 vaccines are an efficient method of reducing both the impact of the disease and the subsequent economic costs; therefore, the worldwide demand for anti-PCV2 vaccines is high [5].

At present, five vaccines against PCV2 have been introduced into the international market. Three of these contain PCV2 capsid protein, which is expressed in baculovirus as an immunogenic virus-like particle antigen, and two contain inactivated PCV2 virions or a PCV2/PCV1 chimera [1]. In all cases, reports from field trials suggest that commercially available PCV2 vaccines make a significant contribution to reduced mortality and improved pig growth on PMWS-affected farms, thereby reducing the economic impact of PCVAD on pig production worldwide [6].

We have long sought to develop an oral PCV2 vaccine for use by the swine industry. Oral administration should be more effective and reduce the indirect costs associated with injectable products. Developing injectable vaccines is laborious, time-consuming and expensive, and their administration is stressful for the animals and the products often induce inflammatory responses at the injection site [7]. On the other hand, oral vaccines, which can be administered via food or water, represent an improvement in antigen delivery technology by enabling farmers to boost an animal’s immunity when it falls outside the protective window. This makes oral immunization procedures better suited for mass administration [8]. However, increasing the mucosal immunogenicity of oral vaccines without compromising safety and tolerability is the holy grail of the vaccine industry [9]. Furthermore, antigen digestion at mucosal sites is a factor that limits successful vaccine development; thus recent studies have aimed to microencapsulate different antigens within natural polymers, such as a chitosan, as a vehicle for the delivery of mucosal vaccines [10, 11].

Chitosan has well-defined properties, including good bioavailability and biocompatibility, low cost, and an ability to open intracellular tight junctions; therefore, it may be a suitable polymer for use as a delivery vehicle for oral vaccines [10]. Moreover, functionalized forms of chitosan have attracted considerable interest due to improved mucoadhesivity, permeability, stability, and controlled/extended antigen release profiles at mucosal sites [11].

Recent evidence shows that oral administration of chitosan microparticles increases mucosal and systemic immune responses [12, 13]. The most recent oral vaccination studies show that antigen-loaded chitosan microparticles gain access to the gut-associated lymphoid tissue (GALT) by passing through the M-cells, which is a key step for inducing immune responses [14, 15]. Therefore, it appears that antigens carried by chitosan microparticles may be targeted specifically to the Peyer’s patches, thereby enhancing both local and systemic immune responses [10].

Here, we combined two strategic approaches to develop an experimental oral vaccine against PCV2. The vaccine was based on yeast-derived PCV2 virus-like particles (VLPs) microencapsulated with chitosan.

The PCV2 VLPs were produced using a synthetic optimized PCV2 *cap* gene sequence, which was expressed in *Saccharomyces cerevisiae* (*S. cerevisiae*). The expression and production of the Cap protein in yeast ensures that it is folded correctly and carries the appropriate post-translational modifications, thereby promoting auto-aggregation and the spontaneous formation of VLPs [7]. In addition to inducing a more efficient immune response, such technology provides a new platform for the production of assembled PCV2 antigens.

Therefore, we set out to show that a new vaccine formulation based on raw yeast extracts containing PCV2 VLPs microencapsulated with chitosan would show immunogenic properties after oral administration. We hypothesized that oral administration of these chitosan microcapsules to mice would result in the delivery of VLPs to the GALT and elicit specific cellular responses against PCV2. A mouse model was used for these proof-of-concept experiments so that we could examine the immunological basis of the cellular response to oral immunization in detail and optimize the adjuvant effects of different chitosan-based formulations.

## Results

### Encapsulating the PCV2 cap protein into chitosan microparticles

**Figure 1:**
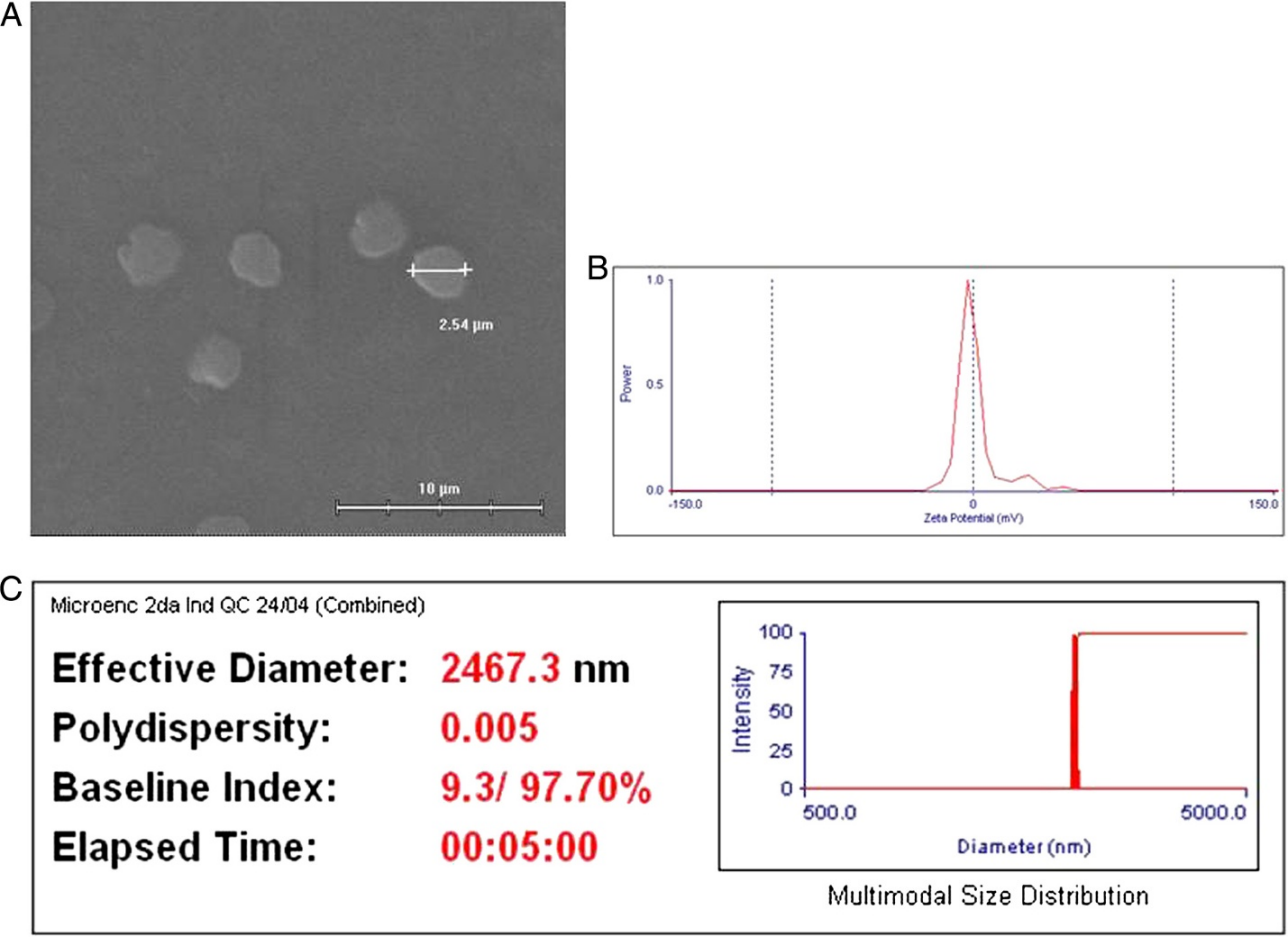
**Chitosan microparticles charged with yeast-derived porcine circovirus type 2 (PCV2) virus-like particles (VLPs) by antigen ionic gelation.** Crude yeast extracts containing PCV2 VLPs were coated with low molecular weight chitosan. **(A)** Particle size and morphology by SEM. **(B)** The Zeta potential was measured to ascertain the average superficial charge (8.19 mV) and **(C)** the overall microparticle size and polydispersity index (0.005) was measured by light scatter analysis.

**Figure 2:**
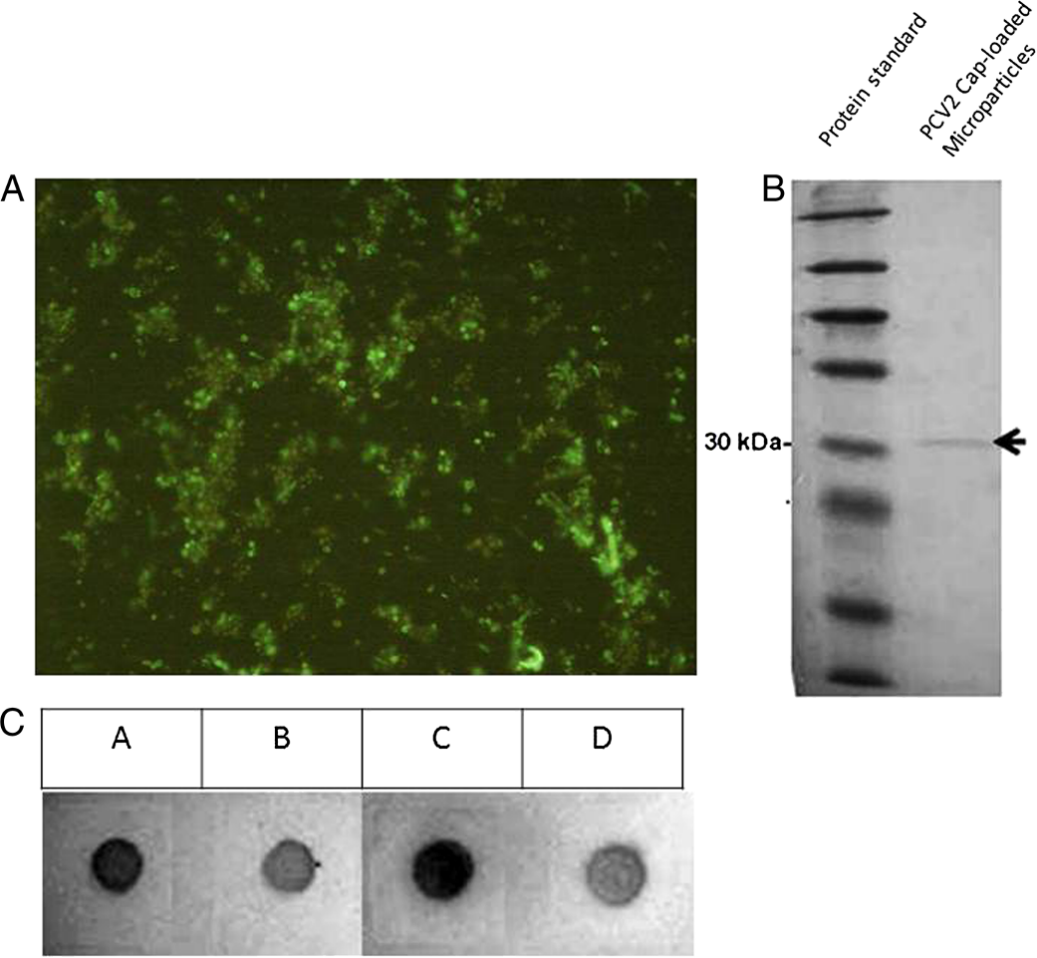
**The porcine circovirus type 2 (PCV2) Cap protein encapsulated in chitosan microparticles.** The PCV2 Cap protein was analysed by **(A)** immunofluorescence microscopy (magnification, ×100), **(B)** Western blotting, and **(C)** Dot blot analysis with an anti-PCV2 monoclonal antibody. For the Western and Dot blots, microparticles were denatured by heating to 90°C for 15 min under acidic conditions. The PCV2 Cap protein contained in each formulation was then detected with a PCV2-specific antibody (Jeno Biotech Inc). PCV2 Cap protein was delivered from purified LMW chitosan-derived microparticles (dots A and C) and from non-purified LMW chitosan-derived microparticles (dots B and D).

**Figure 3:**
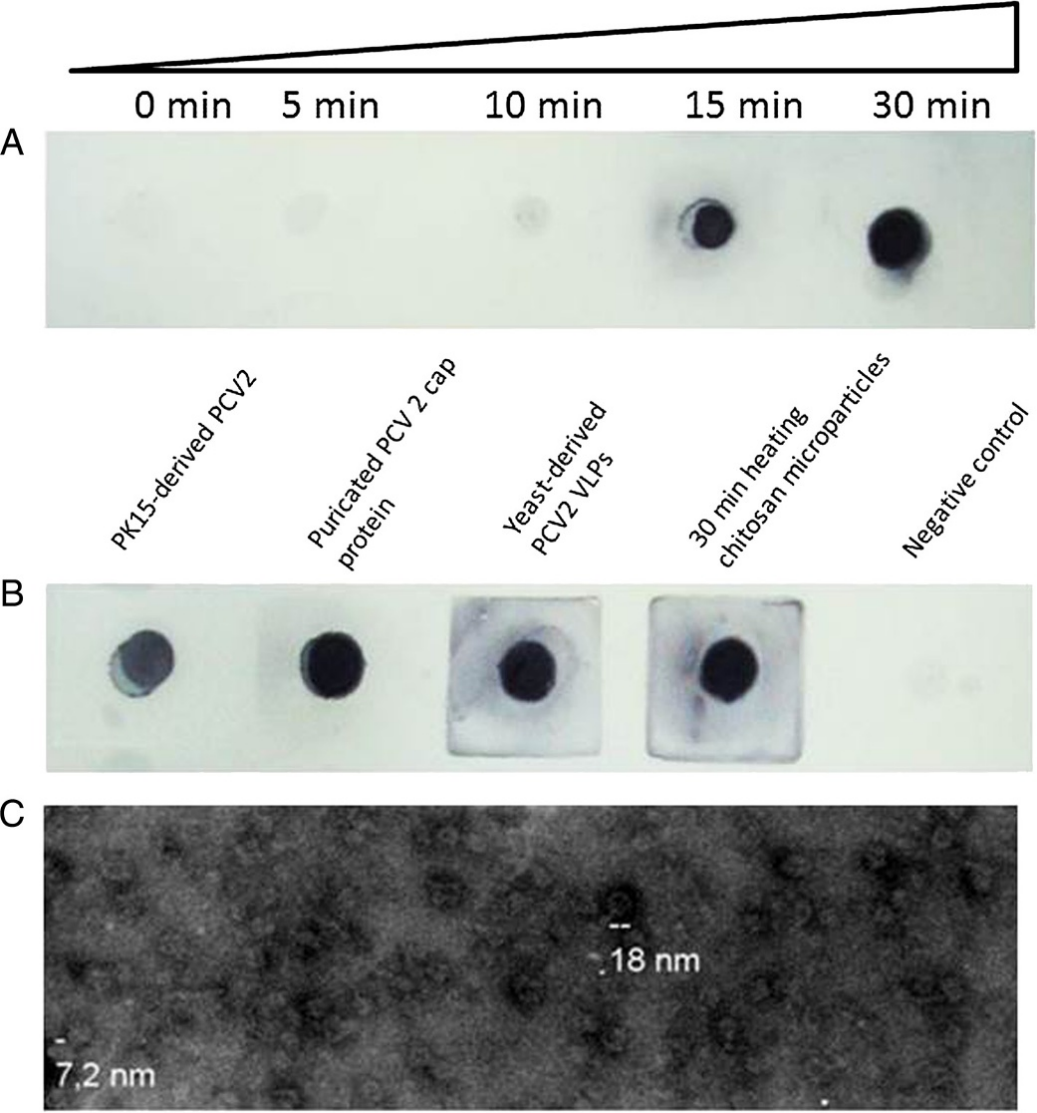
**Presence of the porcine circovirus type 2 (PCV2) Cap protein in the chitosan microparticles. (A)** The kinetics of Cap protein release from chitosan microparticles were analysed by Dot blotting after heating to 90°C for 0, 5, 10, 15 and 30 min under acidic conditions. **(B)** Dot blot analysis was used to estimate the amount of Cap protein loaded into chitosan microparticles relative to purified PCV2 6 × his-cap protein (10 mg/ml). The immunoreactivity of the Cap protein-loaded microparticles was compared with that of PK15-derived PCV2 virions and yeast-derived PCV2 VLPs. **(C)** Electron micrographs showing yeast-derived PCV2 VLPs. The VLPs were viewed using by transmission electron microscopy (Zeiss EM 109) at 80 kV. Magnification, ×85,000.

Crude yeast extracts containing approximately 10% p/p PCV2 VLPs were encapsulated with low molecular weight (LMW) chitosan, which improves both permeability and stability. Scanning electron microscopy showed that the microparticles had an average diameter of 2.5 μm and a spherical morphology (Figure 1A). Analysis of light scatter and Zeta potential revealed that the microparticles had a polydispersity size index of 0.005 and an average superficial charge of 8.19 mV, respectively (Figure 1B and C).Furthermore, purified LMW chitosan showed improved adsorptive affinity and trapping of PCV2 VLPs into microparticles (Figure 2C). The Dot blots show that purified LMW chitosan-derived microparticles (Figure 2C, dots A and C) contained slightly more PCV2 antigen than non-purified LMW chitosan-derived microparticles (Figure 2C, dots B and D).Notably, loading the microparticles with recombinant yeast extracts resulted in their being recognized by anti-PCV2 antibodies, as shown by immunofluorescence analysis (Figure 2A). This suggests that some of the yeast-derived PCV2 antigen was displayed on the surface of the microparticles. Also, we confirmed the presence and integrity of the PCV2 Cap protein in the bulk of chitosan microparticles by denaturing them and immunoblotting the released proteins with an anti-PCV2 antibody. Figure 2B shows that the antibody recognized a specific band of 30 kDa, which is the predicted size of the PCV2 Cap protein, indicating that the Cap protein was not structurally altered during loading into the chitosan microparticles.To determine the maximal amount of antigen that can be captured by a microparticle, we next monitored the release of the Cap protein from the microparticles by heating them under acidic conditions and detecting the release of PCV2 antigen over time (Figure 3A). The Dot blot results showed that maximum antigen release occurred after heating the microparticles at 90°C for 30 min.

Next, we examined whether the yeast extracts to be microencapsulated contained the PCV2 Cap protein in the form of VLPs. Recombinant yeast extracts were purified by sucrose gradient centrifugation and examined under an electron microscope for the presence of nanoparticles, as previously described [7]. The purified yeast extracts contained numerous nanoparticles with an average diameter of 18 nm and a morphology consistent with that of PCV2 VLPs (Figure 3C).

### Chitosan microparticles loaded with yeast-derived PCV2 VLPs elicit antigen-specific cellular immune responses in mice after oral administration

We next examined whether these microparticles could induce the proliferation of antigen-specific lymphocytes isolated from the spleens of C57BL/6 mice after oral administration. For this purpose, splenocytes from orally immunized mice were isolated, re-stimulated with PCV2 virions *in vitro*, and then analysed by flow cytometry. The animals were divided into two groups (n = 3/group). Group 1 was immunized four times with the microparticle formulation (each immunization was separated by a 2 week interval), and the responses were compared with those of the non-immunized control group (group 2).

**Figure 4:**
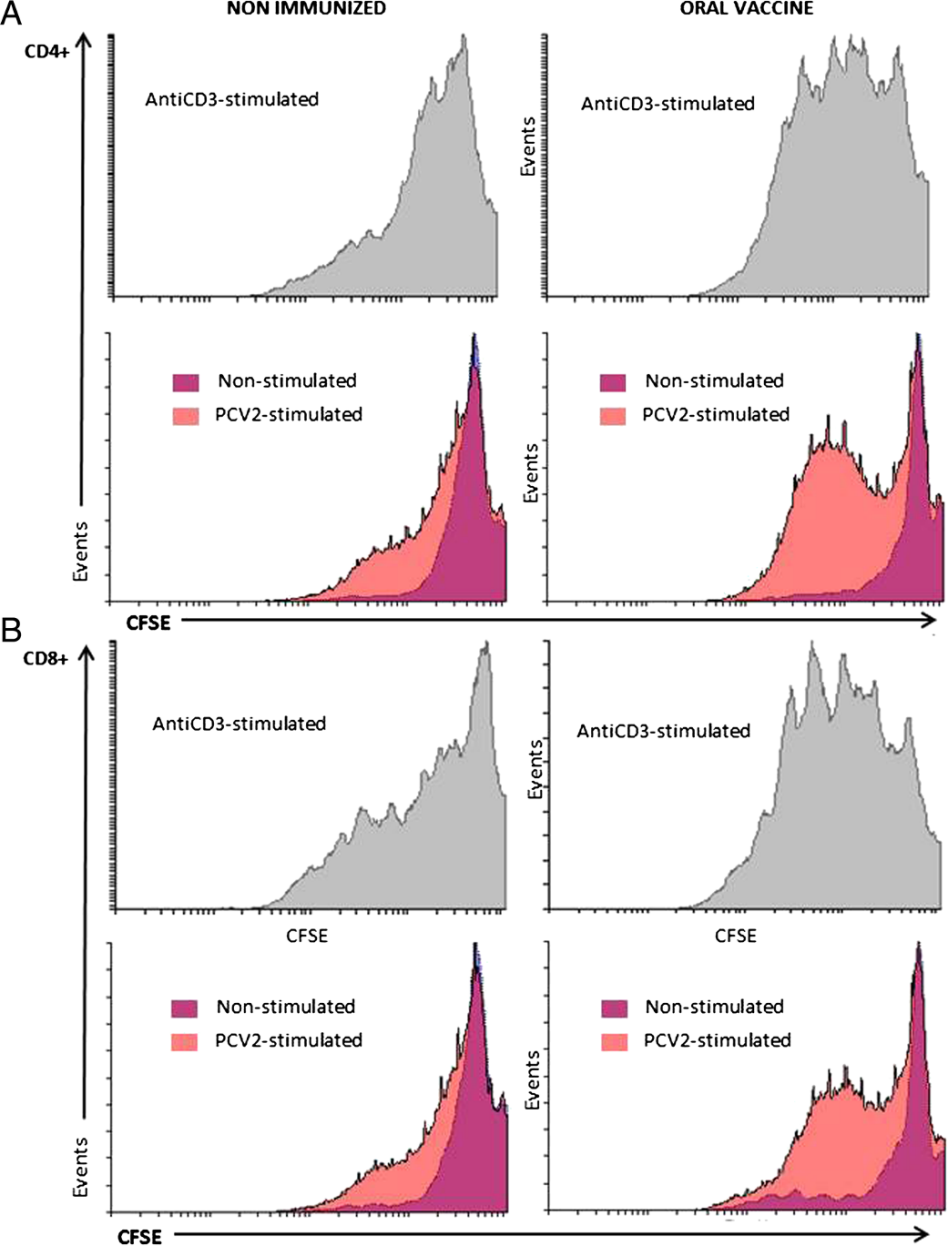
**Murine T-cell responses elicited by immunization with the oral porcine circovirus type 2 (PCV2) vaccine.** The horizontal and vertical axes denote the fluorescence intensity (CFSE) and the number of acquired events, respectively. The CD4^+^ **(A)** and CD8^+^ **(B)** T-cell populations in the spleens of non-immunized mice (right panels) and in the spleens of mice immunized with the chitosan encapsulated vaccine (left panels). Splenocytes were harvested 8 weeks after primary immunization and re-stimulated *in vitro* with PK15-derived PCV2 virions. The cells exposed to PCV2 virions are shown in light red and those not exposed to PCV2 virions are shown in purple (vehicle). As a positive control for non-specific lymphocytic proliferation, splenocytes were incubated in 96-well plates coated with anti-CD3 antibodies (grey histograms). The results show representative histograms from two independent experiments.

Flow cytometric analysis of CD4^+^ splenocytes isolated from immunized mice and re-stimulated *in vitro* with PCV2 virions (Figure 4A, right panel) showed several peaks of low CFSE fluorescence, which is consistent with the presence of cell progeny and suggests PCV2-specific lymphocyte proliferation. Analysis of CD8^+^ splenocytes under the same conditions (Figure 4B, right panel) produced the same result. We also analysed these T-cell populations in non-immunized mice, showing a little difference between the proliferation of cells exposed to the virus and that of non-exposed cells (Figure 4A and B, left panels).

This experiment suggests that splenic T-cell populations (CD4^+^ and CD8^+^) in orally immunized mice actively proliferate upon exposure to the virus. The quantitative data derived from exposed and non-exposed cells inside the proliferation gate for each group is summarized in Table 1.

**Table 1:** **Flow cytometry analysis of splenic CD4+ and CD8+ cells inside the proliferation gate for mice immunized with the experimental oral PCV2 vaccine**

		Oral vaccine	Non-immunized
CD4+	Non-stimulated	29.37 ± 2.04	13.08 ± 4.83
	PCV2-stimulated	48.60 ± 6.92	28.30 ± 8.83
CD8+	Non-stimulated	38.16 ± 5.02	19.39 ± 5.27
	PCV2-stimulated	63.26 ± 1.23	32.95 ± 1.14

The table shows the percentage of CFSE-stained CD4^+^ and CD8^+^ cells (derived from orally immunized and non-immunized mice) present in the proliferation gate after re-stimulation (or vehicle stimulation) with PCV2 virions. Data represent the mean ± standard deviation of two independent experiments.

**Figure 5:**
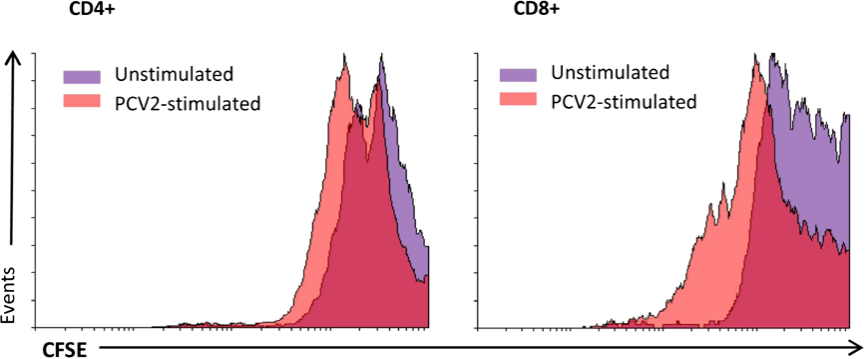
**Murine T-cell responses elicited by immunization with a commercial porcine circovirus type 2 (PCV2) vaccine.** The horizontal and vertical axes denote the fluorescence intensity (CFSE) and the number of acquired events, respectively. The CD4^+^ (left) and CD8^+^ (right) T-cell populations in the spleens of mice immunized subcutaneously with a commercially available PCV2 vaccine, CircoFLEX^TM^. Splenocytes were harvested 8 weeks after primary immunization and re-stimulated *in vitro* with PK15-derived PCV2 virions. Cells exposed to PCV2 virions are shown in light red and those not exposed to PCV2 virions are shown in purple (vehicle). The results show representative histograms from two independent experiments.

An additional control group was used to examine the immune response elicited by a commercial PCV2 vaccine, which was administered subcutaneously. The results showed that, even though the animals exhibited an important base-line proliferative response, the response was equivalent to that of animals immunized with the oral vaccine, particularly in terms of CD8^+^ cells, which showed an important proliferative response until the third and fourth cell generations (Figure 5). These data are quantitatively presented in Table 2.

**Table 2:** **Flow cytometry analysis of splenic CD4+ and CD8+ cells inside the proliferation gate for mice immunized with a commercial anti-PCV2 vaccine**

		Circoflex™	Non-immunized
CD4+	Non-stimulated	31.38 ± 14.08	13.08 ± 4.83
	PCV2-stimulated	50.03 ± 9.71	28.30 ± 8.83
CD8+	Non-stimulated	39.43 ± 9.75	19.39 ± 5.27
	PCV2-stimulated	63.29 ± 13.33	32.95 ± 1.14

The table shows the percentage of CFSE-stained CD4+ and CD8+ cells (derived from mice immunized with CircoFLEX^TM^ as a market anti-PCV2 vaccine and non-immunized mice) present in the gate of proliferating cells after re-stimulation with PCV2 virions or vehicle stimulation. Data represent the mean ± standard deviation of two independent experiments.

### IFN-γ induction after oral immunization

**Figure 6:**
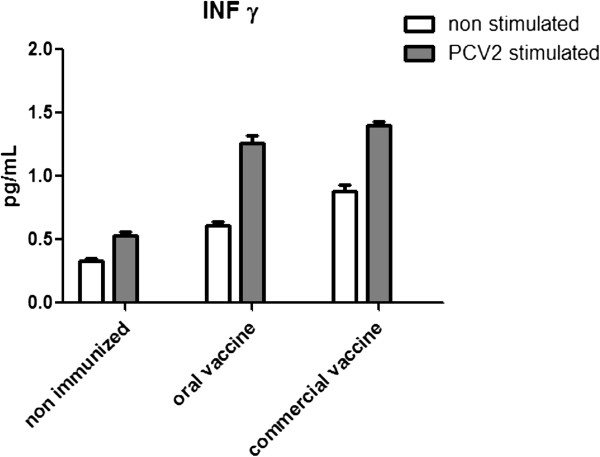
**IFN-γ levels in the supernatants of splenocytes harvested from immunized mice.** Splenocytes were isolated 8 weeks after the first immunization and re-stimulated *in vitro* with porcine circovirus type 2 virions. IFN-γ levels in the supernatant were analysed in a mouse IFN-γ enzyme-linked immunosorbent assay. Data represent the mean ± standard deviation of triplicate wells.

An enzyme-linked immunosorbent assay was used to measure the concentration of murine IFN-γ in spleen cell culture supernatants. Splenocytes isolated from orally immunized mice and re-stimulated *in vitro* with PCV2 virions produced significantly more IFN-γ than splenocytes isolated from non-immunized mice to levels comparable with those induced by an injectable commercial formulation (*p* < 0.01) (Figure 6).

## Discussion

Here, we examined the oral vaccine concept in mice by studying the ability of chitosan-microparticles loaded with minimally purified yeast material enriched with PCV2 VLPs to elicit PCV2-specific cellular immune responses.

We previously showed that *S. cerevisiae* is a simple and safe system in which to generate virus-like PCV2 particles that induce PCV2-specific antibody responses in mice after oral administration [7]. Therefore, we hypothesized that the successful initiation of anti-PCV2 mucosal responses after oral administration of yeast-expressed PCV2 Cap protein would depend on effective antigen delivery to mucosal sites, as well as upon correct folding and self-assembly of the antigen into VLPs. However, the adjuvant effect of the yeast cell constituents must also be taken into account. The latter assertion is supported by several reports showing that yeast cell wall components, mainly β-glucans, stimulate immune responses at mucosal sites [16–20].

On the other hand, the low stability of antigens exposed to the harsh conditions in the gastrointestinal tract, together with the induction of mucosal tolerance, make the induction of a reliable immune response through the oral delivery of assembled viral antigens very difficult. Thus, either large doses of antigens or very stable antigens are required. Therefore, to circumvent these issues, we encapsulated crude yeast extracts containing PCV2 VLPs with LMW chitosan, which improves both cell permeability and antigen stability. LMW chitosan also shows good adsorptive affinity for the PCV2 antigen, enabling it to be efficiently encapsulated into microparticles. Our rationale was based on the hypothesis that chitosan structurally resembles heparan sulfate, a glycosaminoglycan that is the natural receptor for PCV2 on host cells [21]. Also, yeast-derived PCV2 VLPs have a negative Zeta potential (-12 mV, data not shown), which is opposite to that of chitosan microparticles (+8.19 mV); thus the chitosan and the PCV2 VLPs may be electrostatically attracted to each other.

It is also worth considering that part of the yeast-derived PCV2 antigen is located on the surface of the chitosan microparticle, enabling it to react specifically with the anti-PCV2 antibody. Thus, in this case, the microparticle formulation did not follow a core-shell stratification, meaning that the antigen is located within the core and the chitosan simply coats the surface. Therefore, when the antigen is microencapsulated by ionotropic gelation from a homogeneously dispersed polymer-antigen solution, a percentage of the antigen is retained in the matrix of the microparticle and the rest is displayed on the microparticle surface. Thus, the exposed antigen could be detected by immunofluorescence analysis using an anti-PCV2 antibody. This experiment confirmed that the PCV2 antigen was displayed on the microparticle surface (Figure 2A). Similar fluorescent data have been observed for other chitosan microparticle formulations [22].

Previous *in vivo* studies show that coating vaccine antigens with chitosan microparticles is the key to the success of mucosal immunization in mice. For example, van der Lubben et al. carried out extensive research into the use of chitosan microparticles for the delivery of mucosal vaccines and, in particular, their uptake by Peyer’s patches [10, 14, 15, 23]. They generated a human intestinal M-cell model by co-culturing intestinal epithelial cells (Caco-2) and GALT-derived B-lymphocytes (Raji cells), and then investigated the uptake of microparticles [15]. They found that chitosan microparticles were taken up by M-like cells within the artificial epithelium; uptake by Peyer’s patches was also confirmed in a murine model [23]. A common finding of these studies was that the size of microparticles is a key factor: efficient uptake by M-cells requires microparticles measuring < 10 μm in diameter if they are to reach the dome of the Peyer’s patches [24]. Since chitosan is biodegradable, van der Lubben et al. also demonstrated that antigen was released from microparticles after their uptake by M-cells [23]. In general, the behaviour of chitosan microparticles (both *in vitro* and *in vivo*) depends on physicochemical properties such as size, Zeta potential, and surface characteristics [10, 14, 15, 23].

The microparticles formulated and used in the present study measured ~2.5 μm in diameter, and showed very low polydispersity and a slight positive charge. Considering the effect of micro-carrier surface charges on cellular uptake, it is postulated that a positive Zeta potential is beneficial for M-cell transport because the M-cell membrane is negatively charged [25]. In conclusion, both the size and Zeta potential of chitosan microparticles loaded with yeast-derived PCV2 VLPs are key factors that determine access to the GALT, thereby inducing the proliferation of PCV2-specific T-cells and the production of IFN-γ to levels comparable with those induced by an injectable commercial formulation.

This is the first report to explore cell-mediated immune response induced by oral administration of PCV2 antigen encapsulated within chitosan microparticles.

The functionalized (chemically modified) form of chitosan used for preparing microparticles has attracted considerable interest of late due to improved mucoadhesivity, permeability, stability, and a controlled/extended antigen release profile at mucosal sites [11, 26]. Therefore, our future studies will explore the use of these functionalized forms of chitosan for the preparation of microencapsulated PCV2 antigens and their optimization for use as oral vaccines.

## Materials and methods

### Transformation and expression of the PCV2 cap gene in *S. cerevisiae*and production of yeast extracts for microencapsulation

PCV2 cap gene expression was optimized as previously described by Bucarey et al. [7], with some modifications. Briefly, the pYES2::*opt-cap* plasmid was transformed into the expression host, *S. cerevisiae* INVSc1 (genotype: MATα his3Δ1 leu2 trp1-289 ura3-52/MATα his3Δ1 leu2 trp1-289 ura3-; phenotype: His-, Leu-, Trp-, Ura-) using the lithium acetate/single-stranded carrier DNA/polyethylene glycol method [27]. Transformed colonies were cultured in selective autotrophic yeast nitrogen base (YNB) URA ¯ medium (6.7 g of YNB (US Biological, MA, USA), 5 g of casamino acid, 20 g of glucose, 0.03 g of tryptophan (Sigma-Aldrich Co., MO, USA), and 20 g of bactoagar (US Biological, Ma, USA) in 1,000 ml of distilled water) for 48 h at 30°C. The recombinant colonies were picked and transferred into 10 ml of liquid YNB URA ¯ medium and cultured overnight at 30°C until the optical density reached 0.6–0.7 at 600 nm (OD_600_). The cells were then harvested, washed twice with phosphate-buffered saline (PBS), and inoculated into 50 ml of induction medium (YNB URA ¯ medium containing 2% galactose (Sigma-Aldrich Co., MO, USA) instead of glucose) to a final OD_600_ of 0.1–0.3. The cells were then cultured at 30°C with shaking. The cells were induced for 24 h, harvested by centrifugation at 1,500 × g for 5 min at 4°C, and then resuspended in 5 ml of 0.6 M KCl. The cell walls were then digested with liticase (0.1 mg/ml; Sigma-Aldrich Co., MO, USA) at 37°C for 1 h. The resulting protoplasts were sonicated on ice (5 × 60 s cycles with 20 s intervals) using a 102C model Branson Digital Sonifier (Branson Ultrasonics Corporation, CT, USA) operated at 40% amplitude. The raw cell extracts were clarified by centrifugation at 1,500 × g for 5 min at 4°C and analysed by SDS-PAGE followed by Western blotting with a mouse anti-Cap PCV2-specific monoclonal antibody (isotype IgG2a; 1:100 dilution; Jeno Biotech Inc., Republic of Korea), as described by Bucarey et al. [7]. The raw extracts (without clarification) were lyophilized and ground to produce the yeast powder used for microencapsulation into LMW chitosan (75–85% deacetylated; Sigma-Aldrich Co., MO, USA).

### Chitosan and yeast-derived PCV2-antigen microencapsulation

Preparation of the vaccine formulation included the microencapsulation of the viral antigen to protect it and control its release at the mucosal level. Approximately 30 mg of PCV2 Cap protein was used for each microencapsulation. The amount was calculated by assuming that approximately 10% p/p of the dry weight of the recombinant yeast extract comprised PCV2 VLP (data obtained from a previous report [7]). The PCV2 VLP antigen was coated with LMW chitosan (Sigma-Aldrich, MO, USA) by ionotropic gelation as previously described [23], with some modifications. Briefly, 30 ml of LMW chitosan (1% w/v in 2.5% acetic acid) was mixed with 375 mg of dry raw yeast extract (*S. cerevisiae*/pYES2::*opt-cap*) with mechanical stirring (1510 rpm). The microencapsulation reaction was initiated by the drop-wise addition of 5 ml of sodium tripolyphosphate (TPP; 3 mg/ml) (Sigma-Aldrich, MO, USA) at a rate of approximately 1 ml/min (with constant stirring). The solution was then stirred for a further 20 min at room temperature.

The resulting microparticle suspension was centrifuged at 3,000 × g for 10 min. The efficiency of the microencapsulation process was 90–95% (estimated by subtracting the total amount of yeast-derived protein remaining in the supernatant from the initial amount of protein added). Protein concentrations were measured using the BCA™ protein assay kit (Pierce, Rockford, IL, USA). The pelleted microparticles were washed twice with Milli-Q water, lyophilized, weighed, and stored. Samples of the lyophilized microparticles were suspended in PBS (pH 7.0; final concentration, 35 mg/ml) and stored at 5°C. These samples were used to test PCV2 antigen delivery *in vitro* and to induce cellular immune responses in mice following oral administration.

### Scanning electron microscopy, size estimation, and measurement of the Zeta potential of chitosan microparticles

Each microparticle formulation was examined under a scanning electron microscope (SEM; Tesla BS 343 operating at 15 KeV; ×3,300 magnification) to examine the morphology and size of the individual microparticles. Briefly, the freeze-dried microparticles were spread onto metallic discs and gold-coated (20 nm thick) using an EMS-550 automated sputter coater.

The Zeta potential of the chitosan microparticles was measured using a Zeta potential analyser (Zeta plus, Brookhaven Instruments Co., NY, USA). All Zeta potential measurements were determined at 25°C in an electric field of 11.00 V/cm. The size and polydispersity index were determined by light scattering using a multi-angle particle sizing option (90PLUS/BI-MAS, Brookhaven Instruments Co.). A stock solution of each chitosan microparticle sample (1.6 mg/ml in ultra-pure water) was used for both Zeta potential and particle size measurements. Ten millilitres of each solution were mixed with 10 ml of bi-filtered KCl (1 mM in ultra-pure water; pH 7).

### Immunofluorescence microscopy of chitosan microparticles

For immunofluorescence microscopy, 10 mg of freeze-dried chitosan microparticles were blocked overnight in 200 μl of PBS/5% skim milk at 4°C and then incubated overnight at 4°C with a mouse anti-Cap PCV2-specific monoclonal antibody (isotype IgG2a, Jeno Biotech Inc.) diluted 1:100 in PBS/0.1% Tween-20 (PBST). After washing with PBST, the microparticles were incubated with FITC-conjugated goat anti-mouse IgG (H + L) (Kirkegaard & Perry Laboratories Inc.) for 1 h. After further washing, the microparticles were visualized under a Nikon Eclipse E400 fluorescence microscope interfaced to a PC running capture software (Nis-Element Br, Nikon).

Microparticles loaded with extracts of *S. cerevisiae* transfected with an empty plasmid (*S. cerevisiae/*pYES2) were subjected to the same treatment and used as a negative fluorescence control.

### PCV2 antigen loading and delivery efficiency of the chitosan microparticles

The release of PCV2 VLP from the chitosan microparticles was measured in Tris-HCl (pH 1). After antigen loading, the microparticles were resuspended in Tris-HCl to yield a 1% w/v suspension. Samples (200 μl) were then incubated at 90°C with gentle shaking. After 0, 5, 10, 15 and 30 min, the tubes were centrifuged (10,000 × g for 2 min). Samples of the supernatant (100 μl) were taken and the amount of non-bound PCV2 VLP was determined using the Dot blot method. Briefly, samples were transferred onto a nitrocellulose membrane using a Biodot™ microfiltration apparatus (Bio-Rad, CA, USA). The nitrocellulose membrane was then blocked overnight in 5% skim milk at 4°C and then incubated overnight at 4°C with a mouse anti-Cap PCV2-specific monoclonal antibody (isotype IgG2a, Jeno Biotech Inc.) diluted 1:100 in PBS/0.1% Tween-20 (PBST). After washing with PBST, the membrane was incubated with horseradish peroxidase-conjugated goat anti-mouse IgG (H + L) (1:1,000 dilution; Kirkegaard & Perry Laboratories Inc.) for 1 h. After further washing, the signal was detected using 4-chloro-1-naphthol/H_2_O_2_ as directed by the manufacturer (Pierce, Rockford, IL, USA). The concentration of yeast-produced Cap protein was estimated by comparing the signal intensities of the blots with those of known concentrations of a highly purified 6xhis-Cap fusion protein as previously described [7].

### Purification of yeast-derived PCV2 virus-like particles

Clarified yeast extract (500 μl) expressing PCV2 Cap protein was layered onto a discontinuous sucrose gradient (20–50%) and centrifuged at 80,000 × g for 18 h using a Beckman SW-28 rotor. The gradients were fractioned by puncturing the bottom of the centrifuge tube and collecting approximately ten fractions. The fraction densities were determined using a refractometer (32-G110e; Carl Zeiss Jena, Germany). Fractions with a density between 1.2 and 1.27 g/cm^3^ (three fractions in all) were pooled, and the presence of Cap protein was determined by SDS-PAGE. The VLP preparations were dialyzed against PBS and stored at −20°C until visualization by transmission electron microscopy (TEM).

### Purification of the PCV2 6xhis-Cap protein

The complete PCV2 capsid protein gene was subcloned in a pQE80L expression vector (Qiagen, Inc., USA) via the SphI and KpnI restriction sites to generate an in-frame genetic fusion bearing a polyhistidine tag. The bacterial Cap protein was used to produce a purified 6xhis-Cap fusion protein for use as a PCV2 protein standard for blotting as described previously [7]. Briefly, the recombinant *E. coli* strain, BL21 (Amersham), containing the pqE80L::*cap* plasmid was grown in Luria Broth medium (10 g l^-1^ yeast extract, 16 g l^-1^ tryptone, 5 g l^-1^ NaCl, 100 μg/ml ampicillin, pH 7.0) and induced for 5 h at 37°C with isopropylthio-b-D-thiogalactoside (IPTG) at a final concentration of 0.1 mM. The cells were pelleted and resuspended in lysis buffer (8 M Urea, 10 mM Tris, 100 mM NaH_2_PO_4_, 1% Triton X-100, pH 8.0) and then lysed by sonication on ice for two 60 s cycles using a Branson Digital Sonifier® operated at 10% amplitude. After centrifugation at 10,000 × g for 10 min at 4°C, the supernatant was loaded onto a Ni-NTA affinity column (Ni-NTA Purification System, Invitrogen, CA, USA) according to the manufacturer’s protocol. After washing twice with PBS, the Cap protein was eluted in elution buffer (50 mM Tris-HCl, 10 mM imidazole, pH 8.0) and collected. The collected samples were analysed by SDS-PAGE and Western blotting, as described below. The concentration of Cap protein was determined using a Coomassie (Bradford) Protein Assay Kit (Pierce, Rockford, IL, USA).

### Transmission electron microscopy

Yeast-produced VLP preparations (20 μl) were diluted 1/10, adsorbed onto a carbon-coated copper grid, and incubated for 5 min. The grids were then dried using filter paper, negatively stained with 3% phosphotungstic acid (PTA) for 5 min, and viewed using a transmission electron microscope (Zeiss EM 109) operating at 80 kV.

### Animal experiments

Male C57BL/6 mice (5 weeks old) were obtained from the Faculty of Veterinary Sciences at the University of Chile. The animals were assigned to two experimental groups (n = 3 mice/group) and maintained in a temperature and light-controlled environment with access to food and water *ad libitum*. One group (group 1) was used to evaluate specific anti-PCV2 cellular responses against PCV2 virions after oral administration of chitosan microparticles loaded with raw extracts of *S. cerevisiae* expressing the yeast-optimized *cap* gene (*S. cerevisiae/*pYES2::*opt-cap*). The second group comprising untreated (control) mice (group 2) was subjected to the same treatment regimen, but they received PBS alone.

Group 1 received four 200 μl doses of a solution containing 35 mg of chitosan microparticles dissolved in 1 ml of PBS (approximately 7 mg of microparticles per mouse) via oral gavage, with a 14 day interval between doses. The concentration of PCV2 Cap protein in each dose (approximately 300 μg) was determined by densitometric analysis of Dot blots generated using standard dilutions of known concentrations of a bacterially produced Cap-6 × his fusion protein. Group 2 received four 200 μl doses of PBS.

These immunization experiments were repeated twice under the same conditions; thus the total number of animals analysed was 12.

An additional control group (n = 3) was used to examine the immune response elicited by a commercially available PCV2 vaccine (Ingelvac® CircoFLEX™, Boehringer Ingelheim Vetmedica GmbH), which was administered subcutaneously. Briefly, 0.1 ml of formulated vaccine, containing approximately 100 μg of PCV2 antigen, was injected subcutaneously, followed by a booster immunization (with the same dose) 2 weeks later. A third and final immunization was performed 2 weeks after boosting [28].

Animals were sacrificed by an overdose of a mixture of isoflurane/O_2_. The experimental protocol was approved by the institutional animal bioethics committee as stipulated in the guide to the care and use of experimental animals of the Canadian Council on Animal Care.

### Analysis of T-cell proliferation

Animals were euthanized on Day 42 of the experiment as described above, and the spleens were aseptically removed and ground through a sterile cuprous mesh into PBS. A suspension of individual cells was then obtained by repeated passage through a 21G syringe. The splenocytes were then centrifuged and resuspended in erythrocyte lysis buffer (150 mM NH_4_Cl, 10 mM KHCO_3_, 1.3 mM EDTA). After washing with PBS, the cells were stained with CFSE (CellTrace™; CFSE Cell Proliferation Kit, Molecular Probes) as previously described [29], with some modifications. Briefly, cells (5 × 10^7^) were incubated with 1 ml of PBS containing 10 μM CFSE at 37°C for 10 min. The cells were then washed twice with PBS/5% FBS (Foetal Bovine Serum) resuspended (at 2 × 10^6^ cells/ml) in RPMI medium (Thermo Scientific™, MA, USA) supplemented with 10% FBS and then seeded into 96-well plates at a density of 4 × 10^5^ cells/well. The stained splenocytes were then re-stimulated with PCV2 virions (10 TCID_50_ in 50 μl of MEM-α) obtained from PCV2-positive PK-15 cells (ATCC CCL-33) [30]. Non-stimulated splenocytes were used as a negative control (vehicle). As a positive control for non-specific lymphocytic proliferation, splenocytes were incubated in 96-well plates coated with anti-CD3 antibodies (polyclonal stimulators). The cultures were incubated for 96 h and lymphocyte proliferation was examined by acquiring 100,000 events in a FACSCalibur® flow cytometer (Becton Dickinson Immunocytometry Systems, CA, USA). Data were analysed using Flowing Software, version 2.5.

Initially, the population of interest was defined by gating on SSC (cellular complexity) and FSC (cell size). The population of interest was further defined as viable mature and immature lymphocytes, as previously described [31]. This population contained the highest percentage of CD3^+^ cells. Further population analysis was performed by gating on FSC and the CD4 or CD8 lymphocyte markers.

### Measurement of IFN-γ secretion

A mouse IFN-γ enzyme-linked immunosorbent assay (ELISA) Kit (Thermo Fisher Scientific Inc, MA, USA) was used to measure the concentration of IFN-γ in T-cell culture supernatants according to the manufacturer’s instructions. Briefly, culture supernatants from splenocytes derived from immunized and controls mice were diluted 1:50 in PBS, and 100 μL of the resulting solution was added to triplicate wells of the ELISA plate. The absorbance was measured at 550 nm and at 450 nm in a Microplate Reader (Bio-Rad Instruments, CA, USA). The former value was then subtracted from the latter. A standard curve was constructed using a set of standards provided by the manufacturer and the experimental values were read off this curve.

### Statistical analysis

The IFN-γ ELISA assay results were expressed as the mean ± standard deviation. Differences between groups were analysed by ANOVA with Tukey’s post-test. A *p*-value < 0.01 was considered significant. Analyses were performed using GraphPad Prism software.
